# Identification of new miRNA biomarkers associated with HER2-positive breast cancers

**DOI:** 10.18632/oncoscience.275

**Published:** 2015-12-02

**Authors:** Hossam Tashkandi, Nirav Shah, Yogin Patel, Hexin Chen

**Affiliations:** ^1^ Department of Biological Science, University of South Carolina, Columbia, SC, U.S.A; ^2^ Center for Colon Cancer Research, University of South Carolina, Columbia, SC, U.S.A

**Keywords:** microRNA, miRNA, biomarker, breast cancer, HER2

## Abstract

Human epidermal growth factor receptor 2 (HER2) is overexpressed/amplified in ∼30% breast cancers which are associated with poor prognosis. microRNAs are small non-coding RNA which play an important role in many physiological conditions including cancer. Here we screened and identified many miRNAs which are dysregulated by HER2 overexpression. In line with our quantitative PCR analysis data, *in silico* analysis of microRNA expression profiles of 1302 breast tumors revealed that miR-146a-5p is up-regulated and miR-181d and miR-195-5p are down-regulated in HER2-positive tumors. Furthermore, the expression levels of these microRNAs can significantly predict patient survival and thus potentially serve as new prognostic markers for HER2-positive breast cancer.

## INTRODUCTION

Breast cancer is a heterogeneous disease, comprising of multiple entities associated with distinctive histological and biological features. Based upon microarray gene expression profiles, breast cancers can be classified into biologically and clinically meaningful subgroups: luminal A, luminal B, basal-like, normal-like, and human epidermal growth factor receptor 2 (HER2) positive tumors [1]. Luminal A and luminal B breast tumors are estrogen receptor positive (ER+). Among all types of breast cancers, luminal A has the best prognosis for the patient along with the normal-like tumors. Basal-like tumors, that are negative for ER, progesterone receptor (PR) and HER2, are found in 10%-15% of all patients and showed the worst prognosis among all subtypes of breast cancers [2]. HER2 is over-expressed/amplified in approximately 20–30% breast cancers, and this type of breast cancer is known as HER2-positive breast cancer [3]. Major HER2-downstream signaling cascades include phosphoinositide 3-kinase (PI3K)/AKT and Ras/MAPK kinase pathway which are known to regulate cell proliferation and survival [4, 5]. Overexpression of HER2 is associated with relatively poor prognosis and predictive for response to trastuzumab, a monoclonal antibody that targets HER2. However, the average response rate for trastuzumab treatment was approximately 35% with response rate varying from 12–68% [6], necessitating the identification of more biomarkers for selection of treatments and prediction of clinical outcomes.

MicroRNAs (miRNA) are small non-coding RNA that negatively regulate protein-coding messenger RNAs (mRNA) at the post transcriptional level. Dysregulation of miRNAs have been found to be associated with many human diseases including cancer [7]. miRNAs can be oncogenic miRNA or tumor suppressor miRNA depending on their target genes. In addition, miRNAs have also been proposed to be used as biomarkers for cancers, for example, miR-146a-5p as a biomarker for colorectal tumor localization [8, 9]. However, miRNAs' involvement in the HER2 pathway have not studied extensively, and their role in HER2 signaling remains elusive. We propose that the HER2 overexpression will lead to differential regulation of a number of miRNAs which are important in HER2 mediated tumorigenesis.

## RESULTS

### Identification of differentially expressed microRNAs in HER2-overexpressing cells using q-RT-PCR analysis

**Figure 1:**
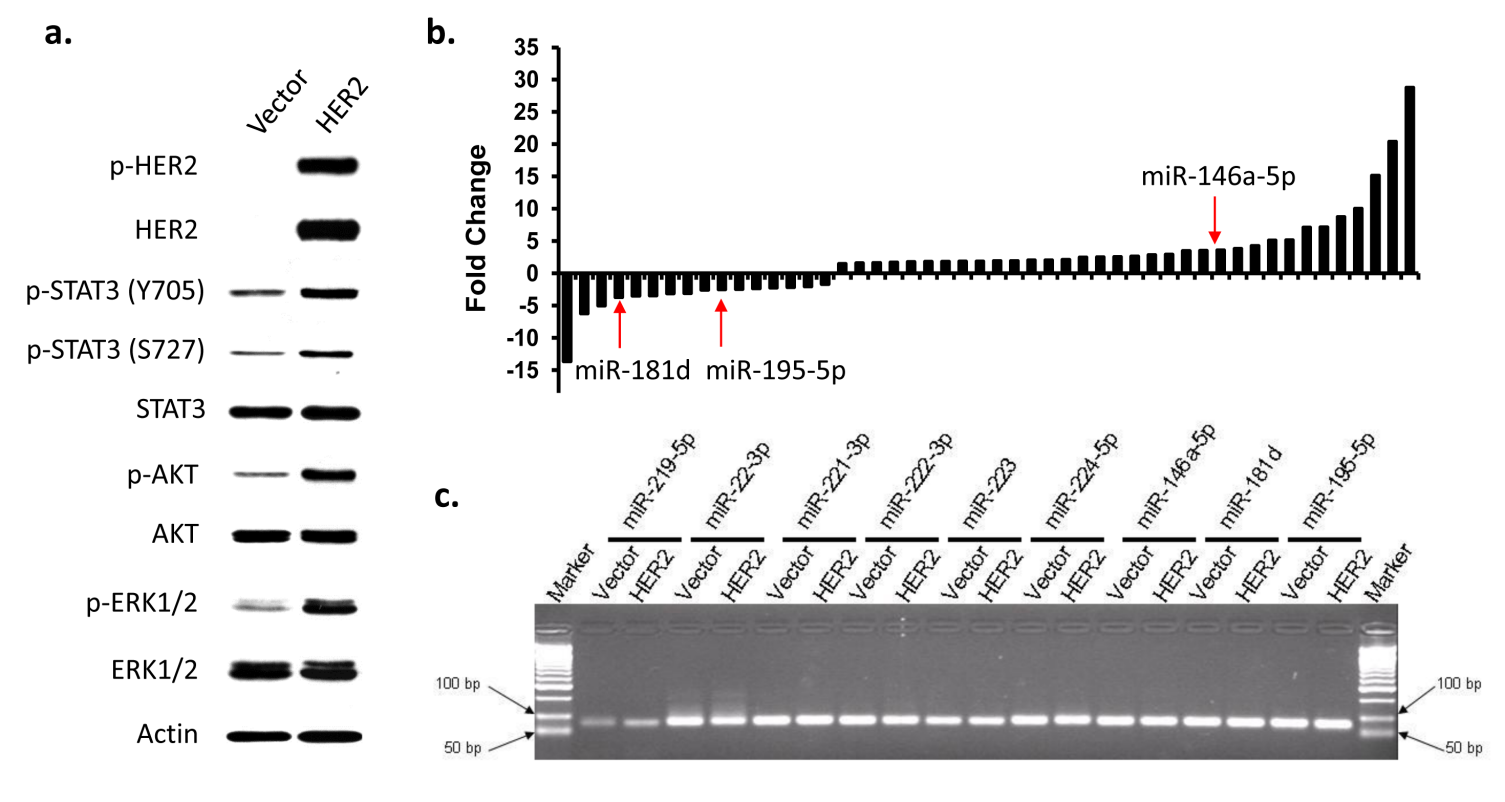
Identification of differentially expressed miRNAs in HER2-overexpressed breast epithelial cells MCF10A-Vector and MCF10A-HER2 were used to screen for differentially regulated miRNAs. a) Western blot analysis of HER2-downstream signaling pathways in MCF10A-Vector and MCF10A-HER2 cells. b) Differentially expressed miRNAs in HER2-overexpressed cells. Over 300 miRNAs were screened using qPCR analysis. The differentially expressed miRNAs were selected with the 2-fold change cutoff. c) Agarose gel picture depicting the expression of representative qPCR products from the miRNA screening.

For screening purposes, immortalized human breast epithelial cells called MCF10A-Vector and its HER2 positive counterpart were used. To confirm whether HER2 transformed cells do produce HER2 receptor, a western blot was performed for evidence (Figure 1a). The MCF10A-HER2 indeed have a higher expression of the phosphorylated HER2 (p-HER2), which signifies the activation of the HER2 receptor in the cells. The activation of HER2 triggers the activation of the downstream signaling pathways. This downstream pathway activation is affirmed by the increased phosphorylation of STAT3 (p-STAT3 Y705 and S727), AKT (p-AKT) and ERK (p-ERK1/2), which are well-known downstream targets of the HER2 signaling pathway [10].

Real-time RT-PCR analysis were performed to screen for miRNAs that are differentially regulated by HER2. Three hundred and three cancer-related miRNA were selected for this screening. Considering that some primers may not work at this PCR condition, the melting curve analysis for each pair of primer was performed and the qPCR products were run on agarose gel to determine if the product of each miRNA primer is correct (Figure 1c). For the qPCR products which didn't show single band and correct sizes or have low abundance (> 35 PCR cycles in both cells) were eliminated for further study. Using two fold change as the cutoff, we further narrowed down the list to fifty miRNAs that are differentially expressed in MCF10A-HER2 cells compared to MCF10A-Vector cells (Figure 1b). Among these differentially expressed miRNAs, miR-146a-5p, miR-195-5p, and miR-181d are potentially involved in breast cancer development based on previous research and therefore they were selected for further statistical analysis.

### *In silico* analysis of miRNAs expression in breast cancer

**Figure 2:**
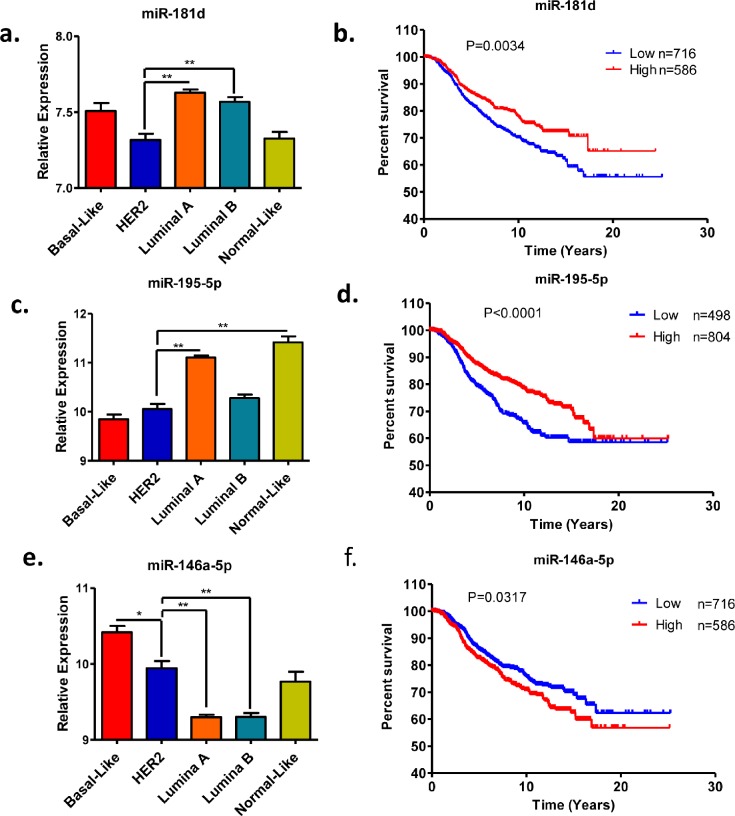
Expression and prognostic values of miR-181d, miR-195-5p and miR-146a-5p in clinical breast cancer samples (a, c, e) Relative expression levels of miR-181d, miR-195-5p and miR-146a-5p in breast cancer subtypes. (b, d, f) Kaplan-Meier survival analysis based on the miR-181, miR-195-5p and miR-146a-5p expression levels. The microarray data was extracted from previous publication [11]. The mean value of miRNA expression levels was used the cutoff to stratify patient into two groups. *p value < 0.05; **p value < 0.01.

Recently, Devinge et al. have reported the miRNA expression profile of 1302 breast cancer samples by microarray analysis [11]. We first analyzed the expression statuses of the three selected miRNAs in the five subtypes of breast cancers. Both miRNAs miR-195-5p and 181d are down-regulated in HER2-positive breast epithelial cells compared to control cells (Figure 1b). Consistently, the expression levels of these two microRNAs are significantly lower in the HER2-positve subtype compared to luminal subtypes of breast cancers (Figure 2a and 2c). However, miR-146a-5p is up-regulated in HER2-positive breast cancer. Indeed, we found that the expression level of miR-146a-5p is higher in HER2-positive and basal subtypes compared to both luminal and normal-like subtypes of breast cancer (Figure 2e). Given that the expression status of these selected miRNAs tend to be associated with the aggressive subtypes of breast cancer, we performed the Kaplan-Meier survival analysis to further evaluate their clinical relevance. As predicted, low expression of miR-195-5p and 181d shows an association with poor patient survival (Figure 2b and 2d) whereas high expression of miR-146a-5p shows an association with poor patient survival (Figure 2f).

## DISCUSSION

Deregulated expression of miRNAs can promote tumorigenesis and contribute to a variety of cancer related phenotypes such as uncontrolled cell proliferation, invasion and metastasis. HER2-overexpressing breast cancers are generally associated with aggressive phenotypes and poor prognosis. Despite of extensive research on HER2 positive breast cancer, the involvement of miRNAs in HER2 signaling pathway is not well understood [12-15]. We screened and identified many miRNAs which were differentially expressed in HER2-overexpressing breast cells compared to control cells. Furthermore, we evaluated their clinical relevance in breast cancer samples. Three miRNAs miR-146a-5p, miR-95-5p and miR-181d show their expected expression profile in HER2-positive breast cancers when compared with other subtypes of breast cancers.

Interestingly, the role of miR-146a in breast tumorigenesis remains controversial. Several studies indicated that miR-146a can function as oncomiR while few have demonstrated it as tumor suppressor miRNA in breast cancer. As an oncomiR, miR-146a could bind to the 3′-untranslated regions (UTRs) of BRCA1 and BRCA2 messenger RNAs (mRNAs) and potentially modulate their mRNA expression [16, 17]. On the contrary, it has been reported that miR146a negatively regulates NF-κB signaling [18, 19]. Overexpression of miR-146a/b results in downregulation of IRAK1 and TRAF6 and subsequently inhibits NF-κB activation, leading to tumor suppression in breast cancer cells [18]. miR-146a has been shown to inhibit both migration, invasion, and metastasis by reducing EGFR expression [20]. It will be interesting to examine the function of miR-146a in the HER2-positive breast cancer.

miR-195-5p has been found to be down-regulated [21] and also been suggested as a possible diagnostic target for breast cancer [22]. Overexpression of miR-195-5p inhibited cell proliferation, reduced cell colony formation, suppressed cell migration and caused an accumulation of cells in the G1 phase of the cell cycle by directly targeting cyclin E1 (CCNE1)[22]. In support of the hypothesis that miR-195-5p acts as a tumor suppressor in breast cancer, we found that miR-195-5p expression is downregulated in aggressive subtypes of breast cancers, especially in HER2-positive and basal types of breast cancers.

Unlike the previous two miRNAs, miR-181d has not been extensively studied. The amount of information and connection between miR-181d and cancer is scarce and it is even more in HER2 positive breast cancers. However, it has been observed that the miR-181 family (miR-181a, miR-181b, miR-181c, and miR-181d), more precisely miR-181c, is activated by HER2 expression [13]. Even though miR-181c is possibly co-expressed or repressed with miR-181d, there has not been many research done regarding this co-expression or repression. Given that miR-181d is down-regulated with the overexpression of HER2 in both cell lines and clinical samples, miR-181d may be classified as a tumor suppressor miRNA and may target an oncogene.

In summary, we presented a strategy to effectively identify novel and clinically relevant microRNA biomarkers by combining a high-throughput screening approach with *in silico* data-mining analysis. Further research is warranted to explore their functions in breast tumorigenesis and develop them as novel therapeutic targets.

## MATERIALS AND METHODS

### Cell culture

MCF10A-Vector and MCF10A-HER2 cells were provided through the courtesy of Emily Wang at the Institute of City of Hope. Culturing MCF10A cells require DMEM/F12 medium from Corning cellgro™. In a 500ml bottle, 25ml of 5% horse serum from Sigma® Life Science is added to the medium along with 100μl of 500μg/ml of cholera toxin for a final concentration of 100ηg/ml. In addition, 1ml of 5mg/ml insulin for a final concentration of 10μg/ml, 63μl of 4mg/ml of hydrocortisone for a final concentration of 0.5μg/ml, and 50μl of 200μg/ml of EGF for a final concentration of 20ηg/ml are added. When detaching cells from cell culture plate, 0.25% of Trypsin acquired from Sigma® Life Science.

### Western blot analysis

Protein extraction was achieved using standard protocol [23, 24]. The protein concentration is then quantified and normalized to ensure equal loading into polyacrylamide gels. 8% polyacrylamide gels were used to separate the proteins. Antibodies used were acquired from Cell Signaling Technology®.

### Real-time RT-PCR analysis of miRNA expression

RNA extraction from this point was done using TRIzol® protocol [25]. RNA was first poly-adenylated using *E.coli* poly(A) polymerase from New England Biolabs. Poly-adenylated RNA was then used for reverse transcription and the reverse-transcription polymerase chain reaction (RT-PCR). For the quantitative polymerase chain reaction (qPCR), 2μl of cDNA was mixed with 10μl of RT^2^ SYBR® Green ROX qPCR Mastermix from Qiagen, 6μl of RNase-free water, 1μl of a universal reverse primer (5′GCG AGC ACA GAA TTA ATA CGA C3′) and a forward primer specific to the miRNA. The reaction is run in an Applied Biosystem™ qPCR machine with the following parameters: Stage 1: 3 minutes hot start at 95°C. Stage 2: 15 seconds denaturing step at 95°C followed by 30 seconds annealing step at 60°C and finally the extension step is held for 35 seconds at 70°C. This stage is repeated for 45 cycles.

### *In silico* analysis of miRNAs expression in breast cancer

The clinical effect of the gene expression profiles of microRNA was evaluated using a published data set of breast cancer patients [11]. The mean expression value was used as the cutoff to classify miRNA expression as high or low. Recurrence-free survival was estimated using the Kaplan-Meier method and compared with log-rank tests. The statistical analysis was conducted with R and GraphPad software packages (GraphPad, CA, USA). The p-values were calculated using the logrank test, and differences were considered statistically significant at p < 0.05.
